# Supplementary material for: Content-rich biological network constructed by mining PubMed abstracts
Source: BMC Bioinformatics. 2004 Oct 8;5:147. doi: 10.1186/1471-2105-5-147 (PMC528731; doi:10.1186/1471-2105-5-147)
Supplement: Additional File 2 — The original results of the above study (non-essential files are deleted to keep the file size under the limit set by BMC bioinformatics). [file 1471-2105-5-147-S2.bz2 › chilibotAdditionalFile2/dip20/137ID7816143E492/html/FOS_JUN.html]

 


 **FOS** and **JUN** 
  
Found 4259 abstracts in PubMed, retrieved 20.  
 

 What does Google say? 
 PDF only 
| .edu only 

---

**Interactive relationship** (e.g. stimulation, inhibition, etc)

**Inhibitory relationship**- Gene expression for  **Fos**  was suppressed at both times, while that for Fosl1 and  **Jun**  was augmented at 12 h and suppressed at 48 h.  Ref: 12875578 Acta Otolaryngol, 2003
**Stimulatory relationship**- FGF2 treatment also induced  **fos**  and  **jun**  mRNAs and later increased the nuclear levels of activator protein 1  [ **JUN** ]  AP1  [ **JUN** ] .  Ref: 12815619 Dev Dyn, 2003
**Neutral relationship**

**Non-interactive relationship** (e.g. studied together, co-existance, homology, etc.)

- In addition to shock response genes, such as hsp70 and hsp40, the stress response genes c  **jun** , c  **fos**  and egr 1 were expressed in the early phase after heat shock.  Ref: 12841876 Cancer Sci, 2003
- Western blots for AP 1  [ **JUN** ]  and its signal mediators Erk and JNK showed that expression of  **Fos**  and JNK were decreased by the addition of FZFAT at 300 microg ml.  Ref: 12856862 Am J Chin Med, 2003
- Supershift electrophoretic mobility shift assay indicated that JunB, JunD, and c  **Fos**  components of AP 1  [ **JUN** ]  were particularly affected.  Ref: 12857751 J Biol ChemJ Biol Chem, 2003
- In addition, using an in vitro model of ovarian cancer, we demonstrated that malignant transformation of ROSE cells resulted in alteration of downstream effectors of the EGFR pathway, as exemplified by aberrant expression of p66Shc, c  **Jun** , c Myc, c  **Fos** , Lot1, p21Cip Waf, and cdc25A.  Ref: 12849999 Biochem Biophys Res Commun, 2003
- IR was found to enhance the expression of c  **Jun**  and, in particular, ATF3, but, in contrast to various other stress stimuli, did not induce the expression of c  **Fos** .  Ref: 12833146 Oncogene, 2003
- MATERIAL AND METHODS From cDNA microarray screening of those specimens, several gene clusters, including Nos2 and the transcription factors  **Fos** , Fosl1,  **Jun**  and Nfkb1, were identified as possible upstream regulators of Na transport protein expression.  Ref: 12875578 Acta Otolaryngol, 2003
- These results demonstrate that contextual memory retrieval is associated with de novo synthesis of specific members of the  **Fos**   **Jun**  transcription factor family.  Ref: 12882314 Genes Brain Behav, 2003
- Using specific polyclonal antisera against c  **Fos** , JunB, c  **Jun**  and JunD, we tried to identify the candidate transcription factors of the immediate early gene family which may contribute to the molecular processes during contextual memory reconsolidation.  Ref: 12882314 Genes Brain Behav, 2003
- CHOP dimerizes with and inhibits the binding of C EBP related transcription factors to their consensus DNA target sequences and also forms novel complexes with other transcriptional proteins e.g. c  **jun** , c  **fos** .  Ref: 12876286 J Biol ChemJ Biol Chem, 2003
- Oncogenes, such as c myc, c  **fos** , and c  **jun** , are also up regulated in this model.  Ref: 12855652 Clin Cancer Res, 2003
- Also, GLP 2 produced a significant increase in the mRNAs of c  **fos**  and c  **jun**  when gene expression was determined by Northern blotting.  Ref: 12846833 Eur J Biochem, 2003
- We first assessed the effect of oxidant exposure on AP 1  [ **JUN** ]  c  **Jun**  and  **Fos**  and c  **Jun**  N terminal kinase JNK regulation in MLE12 cells.  Ref: 12842852 Am J Respir Cell Mol Biol< MedlineTA>Am J Respir Cell Mol Biol, 2003
- A nuclear run on assay showed induction of c  **fos**  and c  **jun**  transcripts at the end of the exposure, peaking at 12hr after resuspension of cells in drug free medium.  Ref: 12818361 Biochem Pharmacol< MedlineTA>Biochem Pharmacol, 2003
- Both VPA and lithium altered the expression of the early inducible genes for c  **fos**  and c  **jun**  thus promoting the expression of specific proteins.  Ref: 12890311 Int J Neuropsychopharmacol, 2003
- The inhibition of S phase induction was associated with down regulation of c Myc, but not of c  **Fos**  or c  **Jun** .  Ref: 12883011 Proc Natl Acad Sci U S AProc Natl Acad Sci U S A, 2003
- In these mice context dependent memory retrieval evoked a marked induction of c  **Fos**  and JunB, but not of c  **Jun**  and JunD, in pyramidal CA1 neurons of the dorsal hippocampus.  Ref: 12882314 Genes Brain Behav, 2003
- Several putative transcriptional factor binding sites for nuclear factor of activated T cells 1, AP1  [ **JUN** ]  c  **JUN**  and c  **FOS** , AP2, GABP, OCT1, GATA3, PRE, and C ETS1 were predicted in the promoter region.  Ref: 12816724 Neuro oncol< MedlineTA>Neuro oncol, 2003
- Following a 24 hr exposure to 100& ; mgr ; M oltipraz, elevated steady state levels of mRNA for  **Jun**  and  **Fos**  family members were observed.  Ref: 12818361 Biochem Pharmacol< MedlineTA>Biochem Pharmacol, 2003
- Both oxidants induced c  **Jun**  and  **Fos**  expression, albeit with a different pattern of regulation hyperoxia 95% O2 inducing a biphasic response while H2O2 500 micro M induced a sustained response.  Ref: 12842852 Am J Respir Cell Mol Biol< MedlineTA>Am J Respir Cell Mol Biol, 2003
- Supershift analysis verified the participation of  **Jun**  and  **Fos**  in the complexes.  Ref: 12818361 Biochem Pharmacol< MedlineTA>Biochem Pharmacol, 2003
- The expression levels of heat shock protein 70 HSP70, c  **jun**  and c  **fos**  genes were remarkably up regulated and those of c myc and poly ADP ribose polymerase PARP were down regulated in bupivacaine treated cells.  Ref: 12820540 J Toxicol Sci, 2003
